# Chromosomal analyses of human giant diploid oocytes by next‐generation sequencing

**DOI:** 10.1002/rmb2.12378

**Published:** 2021-03-18

**Authors:** Hiroomi Kawano, Naoki Yamashita, Junya Ito, Naomi Kashiwazaki

**Affiliations:** ^1^ Laboratory of Animal Reproduction Graduate School of Veterinary Science Azabu University Sagamihara Japan; ^2^ Yamashita Shonan Yume Clinic Fujisawa Japan

**Keywords:** cell size, cytodiagnosis, high‐throughput nucleotide sequencing, micromanipulation, oocytes

## Abstract

**Purpose:**

Although giant oocytes (GOs) having about twice cytoplasmic volume compared with general oocytes in mammals including the human are rarely recovered, it is thought that GOs have potentially chromosomal abnormalities. The aim of the present study was to assess chromosome numbers in chromosome‐spindle complexes (CSCs) and polar bodies of human GOs by using micromanipulation for sampling and next‐generation sequencing (NGS) for analyses of the chromosome numbers.

**Methods:**

When recovered oocytes whose cytoplasm has lager than 140 µm or above, the oocytes were defined as GOs, and recovered GOs were vitrified. After warming, the CSCs, polar bodies, and enucleated cytoplasm were collected by micromanipulation from 3 GOs. The collected samples were analyzed by NGS.

**Results:**

Chromosomal aneuploidy in the GOs was confirmed in all the three GOs. Comparing the CSCs with the chromosomes from polar bodies, the deletion and overlapping chromosome numbers were complementary in each GO.

**Conclusions:**

The authors could collect the CSCs and the polar bodies from human GOs by micromanipulation, and then could analyze the chromosome numbers of the GOs by NGS method. As our data suggest that human GOs have chromosomal abnormalities, GOs should be excluded from clinical purpose as gamete sources for embryo transfer in the human.

## 1 INTRODUCTION

Although giant oocytes (GOs) having about twice cytoplasmic volume compared with general oocytes in mammals including the human are rarely recovered, it is thought that GOs have potentially chromosomal abnormalities. The recovery rates of human GOs account for 0.12%‐0.3% of recovered oocytes.^1^, ^2^, ^3^, ^4^ It is thought that the recovery rates of GOs do not depend on the ovarian stimulation methods or the constitution of the patients.^1^ In general, mature GOs have one or two spindles and polar bodies, and GOs have potentially chromosomal abnormalities.^1^, ^3^, ^4^, ^5^, ^6^ Therefore, although GOs have ability to fertilize in vitro and develop to the blastocyst stage in vitro,^4^ GOs are not used for clinical purposes as sources for embryo transfer. Together with previous reports on human GOs,^3^, ^4^ almost GOs have two polar bodies and chromosome‐spindle complexes (CSCs) as metaphase Ⅱ plates in the cytoplasm. Thus, it is thought that GOs are derived from cells with lack of cytokinesis during mitotic divisions of the oogonia, or cell fusion of two adjacent oogonia or oocytes during oogenesis.^7^ In addition, it is important for fertilization, reproduction, and development to examine chromosome numbers in mammalian gametes including GOs.

In general, cytogenetic analyses of chromosome numbers are needed to collect a lot of cells at metaphase and special staining. Therefore, it is difficult to assess the chromosome numbers in oocytes. Recently, next‐generation sequencing (NGS) and the related technology have been developed with remarkable progress in DNA sequence technology, and then, we could apply it to chromosome analyses, aneuploid screening of embryos.^8^ On the other hand, micromanipulation for oocytes/embryos in mammals has already been developed well, a lot of genetically modified and cloned animals was generated,^9^ and tremendous babies were also born via micromanipulations such as intracytoplasmic sperm injection (ICSI) worldwide. In the present study, we applied these novel techniques to human GOs that are valuable and unique materials to clarify numerical chromosomal constitution during oocyte maturation.

The aim of the present study was to assess chromosome numbers in CSCs and polar bodies of human GOs by using micromanipulation for sampling and NGS for analyses of the chromosome numbers.

## 2 MATERIALS AND METHODS

### 2.1 Oocyte recovery

When patients agreed to provide GOs for research purposes by the informed consent form, recovered GOs were used in the present study. Oocyte recovery was performed at natural cycles or minimal ovarian stimulation (by clomiphene citrate or aromatase inhibitor) cycles in Yamashita Shonan Yume Clinic in Kanagawa, Japan, and recovered GOs at that time were then cryopreserved. The GOs were used for chromosome assessments in the present study.

Oocyte recovery in natural cycles was conducted as follows: When the diameter of dominant follicle reaching 15‐18 mm by transvaginal ultrasound examination and the plasma estradiol (E2) value reached around 250 pg/mL, oocyte maturation was induced by nasal spray of buserelin acetate (Buserecur; Fuji Pharma Co., Ltd.). Then, oocyte recovery was carried out 34 hours after the induction of oocyte maturation. Oocyte recovery in minimal ovarian stimulation cycles was conducted as follows: Clomiphene citrate (Clomid®; Fuji Pharma Co., Ltd.) or aromatase inhibitor (letrozole; Nichi‐Iko Pharma Co., Ltd.) was administered orally for 5 days from the third day of menstruation; depending on the plasma E2 and follicle‐stimulating hormone (FSH) values at the 7th‐8th day of menstruation and the growth follicle diameter and its number, recombinant follicle‐stimulating hormone (Gonal‐f®75; Merck Biopharma Co., Ltd.) was administered. Plasma E2 value, luteinizing hormone (LH), and progesterone (P4) were measured to confirm sufficient follicular development and LH surge. When the dominant follicle size became more than 18 mm, the final oocyte maturation was induced by nasal spray of buserelin acetate, and oocyte recovery was carried out after 34 hours after the induction of oocyte maturation. Oocyte aspiration was performed from follicles reached more than 12 mm in diameter using a 21‐gauge follicle aspirating needle (Kitazato Biopharma) under guided transvaginal ultrasound (SONOVISTA FX, Siemens Healthcare KK).

### 2.2 GO collection and vitrification

Obtained oocyte‐cumulus complexes were treated to remove cumulus cells while leaving a few layers and observed under an inverted microscope (IX‐73; Olympus Corporation, Tokyo, Japan) to check oocyte maturation and measure its cytoplasm size in diameter. Regarding the size of recovered oocytes, those with cytoplasm diameters exceeding 140 µm were judged as GOs.^4^, ^10^ Further cumulus cells were removed by repeated pipetting in 80 IU/mL hyaluronidase (Cooper Surgical, Inc) at 37.0°C.

To confirm oocyte maturation (metaphase II stage), nuclei including CSCs and polar bodies were observed under a polarized light microscopy (IX‐73‐SLICSI; Olympus Corporation). Collected GOs were vitrified ^11^ by using Vitrification kit (VT601, VT602, Cryotop®; Kitazato Corporation) until sampling micromanipulation. First, GOs were inserted into 30 µL of basic solution (BS, HEPES within Basic Culture Medium added hydroxypropyl cellulose and gentamicin) at room temperature around 24°C. Then, the same amount of equilibration solution (ES, HEPES within Basic Culture Medium added 7.5% ethylene glycol, 7.5% dimethyl sulfoxide, hydroxypropyl cellulose, and gentamicin) was added to BS and equilibrated for 3 minutes. Further 30 µL of ES was then added to the droplet included GOs and equilibrated for 3 minutes. Subsequently, GOs were transferred to 300 µL of ES, equilibrated for 9 minutes, and then transferred to 300 µL of vitrification solution (VS, HEPES within Basic Culture Medium added 15% ethylene glycol, 15% dimethyl sulfoxide, 0.5 mol/L sucrose, hydroxypropyl cellulose, and gentamicin). After the transfer for 1 minute, GO was placed on a sheet of a Cryotop®, and the device was then plunged into liquid nitrogen directly. The vitrified GOs were stored in liquid nitrogen until micromanipulation for sampling.

The top of Cryotop® including vitrified GOs was immersed in 2 mL of warming solution (TS, HEPES within Basic Culture Medium added 1.0 mol/L sucrose and gentamicin) at 37°C for 1 minute. After warming, GOs were transferred to 300 µL of diluent solution (DS, HEPES within Basic Culture Medium added 0.5 mol/L sucrose, hydroxypropyl cellulose, and gentamicin) and kept for 3 minutes. After the dilution, GOs were transferred to washing solution (WS, HEPES within Basic Culture Medium added hydroxypropyl cellulose and gentamicin) for 5 minutes and then transferred to Quinn's Advantage^TM^ Protein Plus Cleavage Medium (Cooper Surgical, Inc). The vitrified/warmed GOs were cultured until micromanipulation for sampling in incubators at 37.0°C in the atmosphere of 6% CO_2_, 5% O_2_, and 89% N_2_.

### 2.3 GO micromanipulation for sampling polar bodies and CSCs

**FIGURE 1:**
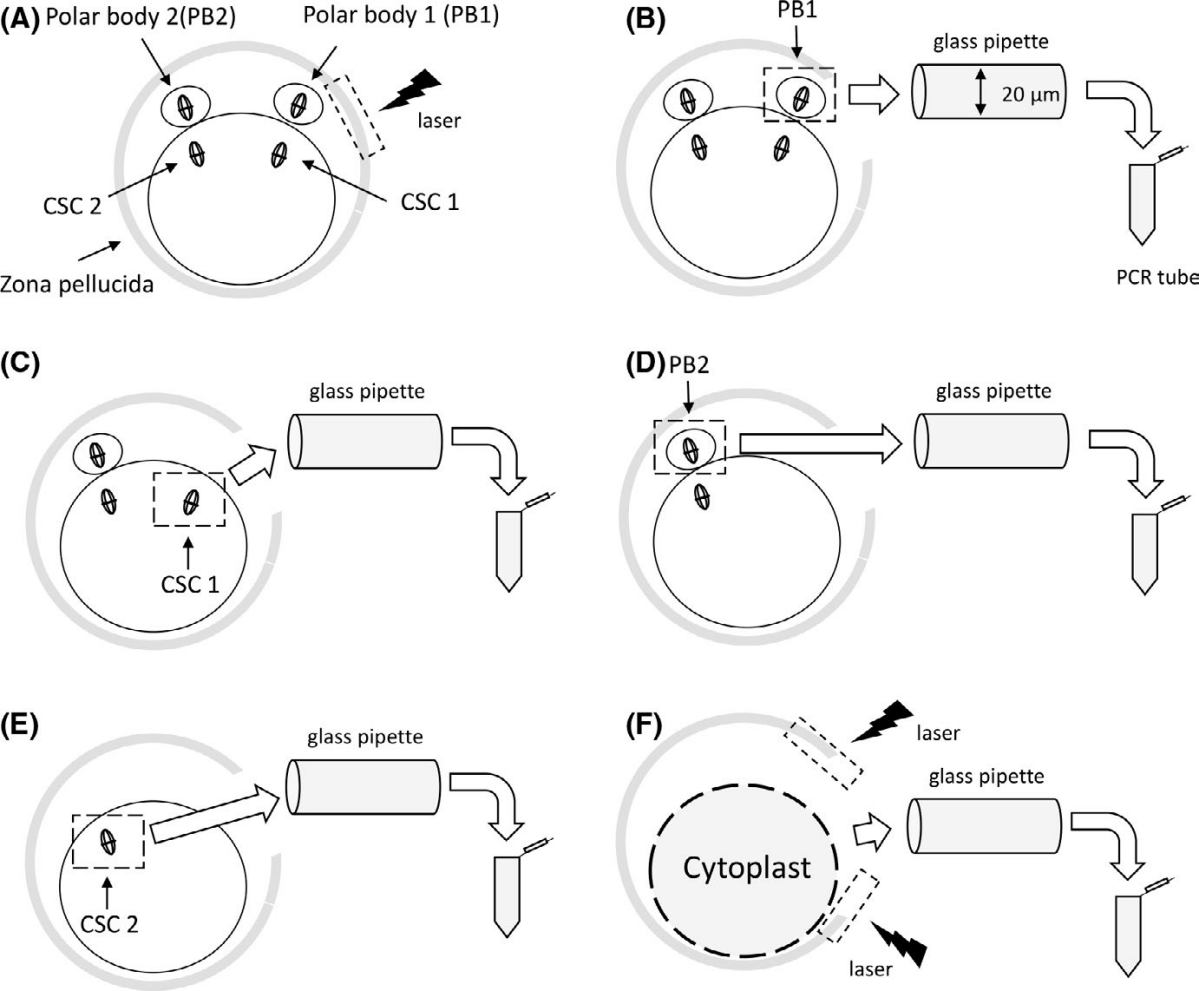
Micromanipulation procedure for sampling polar bodies and chromosome‐spindle complexes (CSCs) from a mature giant oocyte in the human. A, Laser perforation of the zone pellucida. B, Tubing polar body 1. C, Tubing CSC 1. D, Tubing polar body 2. E, Tubing CSC 2. F, Tubing cytoplast. CSC(s), Chromosome‐spindle complex(es)

To avoid the effects of in vitro maturation culture, we only used mature GOs at oocyte recovery. Vitrified and warmed GOs were cultured for 10 minutes, as a pre‐treatment for micro‐manipulation for sampling. Quinn's Advantage^TM^ Protein Plus Cleavage Medium added cytochalasin B (C6762‐5MG; Sigma‐Aldrich) to 5 µg/mL was used for the culture before the sampling micromanipulation for 10 minutes to improve cell viability after micro‐manipulation.^12^ As micro‐manipulation medium (M‐medium), Quinn's Advantage Medium with HEPES (Cooper Surgical, Inc) added 10% plasma protein fraction (PPF, KENKETU ALBUMINATE 4.4% for IV injection 11 g/250 mL, Nihon Pharmaceutical Co., Ltd.) and 5 µg/mL cytochalasin B were used for the micro‐manipulation. A plurality of 10 µL drops of M‐medium and a few drops of M‐medium including 10% (v/w) polyvinylpyrrolidone (PVP) were prepared in the glass bottoms dishes (P50G‐0‐30‐F.I/H, MatTek Corporation) for the micro‐manipulation. The GOs were then transferred to a 10 µL droplet of M‐medium under paraffin oil (OVOIL, Vitrolife) in the glass bottom dishes and micro‐manipulated for collecting polar bodies and CSCs of GOs. To collect individual genomic DNA from polar bodies and CSCs, GOs were individually transferred into a micromanipulation drop, the positions of CSCs in GOs were confirmed with a polarized light microscopy, and a hole with a width of about 20 µm in the zona pellucida of the GO near the polar body was made by an infrared diode laser (Saturn 5TM Laser system; CooperSurgical, Inc; Figure 1A). As shown in Figure 1B, the polar body 1 was aspirated with a glass pipette (PIN20‐20FT; PRIME TECH LTD.) with an inner diameter of 20 µm and transferred to a PCR tube (K77301; BIOplastics) containing 2 µL of phosphate‐buffered saline (PBS), and frozen and stored in a freezer at −30°C up to the chromosome analyses. As shown in Figure 1C, the CSC 1 corresponding to the polar body 1 was aspirated with the pipette, transferred to another PCR tube, and frozen and stored. One set of polar body 1 and CSC 1 derived from the same GO were collected as mentioned above, and the remaining polar body 2 and CSC 2 derived from the same GO were also collected each PCR tubes in the same procedure (Figure 1D,E). After collecting two sets of polar bodies and CSCs derived from the same one GO, the infrared diode laser was applied to the zona pellucida and a hole with a width of about 100 µm was made, and aspirated cytoplast remaining in the zona pellucida from the hole. The cytoplast was transferred to a PCR tube and frozen and stored up to the chromosome analyses (Figure 1F). We sent collected genomic samples that were derived from polar bodies, CSCs, and cytoplasts to Igenomix Japan KK to examine the chromosome numbers of the GOs by NGS analyses.

### 2.4 Chromosome analyses of GOs by NGS

The collected polar bodies, CSCs, and cytoplasts in the tubes were sent to Igenomix Japan KK for NGS analysis to examine the chromosome numbers. The protocol for this NGS analysis was as follows (citation: Report preimplantation genetic testing for aneuploidy PGT‐A, Igenomix Japan KK): The test was conducted by using the Ion ReproSeq^TM^ PGS KIT (Next‐Generation Sequencing) for 24 chromosomes (22 autosomal and X, Y chromosomes) aneuploidy screening (Thermo Fisher Scientific). The Kit/assay was performed on the Ion Chef^TM^ Ion S5 and Ion SS System instruments (Thermo Fisher Scientific). Data analysis was performed with Ion Reporter software, which aligns the reads using the last human genome build (hg19; Thermo Fisher Scientific).

### 2.5 Statistical analysis

GO cytoplast size was measured to two decimal places, and the mean was shown in mean ± SE. Statistical analysis of the GOs recovery rate between ovarian cycles was determined by one‐way ANOVA followed by Tukey's test for multiple comparisons. Significance difference was set at *P* < .05.

## 3 RESULTS

### 3.1 Oocyte recovery

**FIGURE 2:**
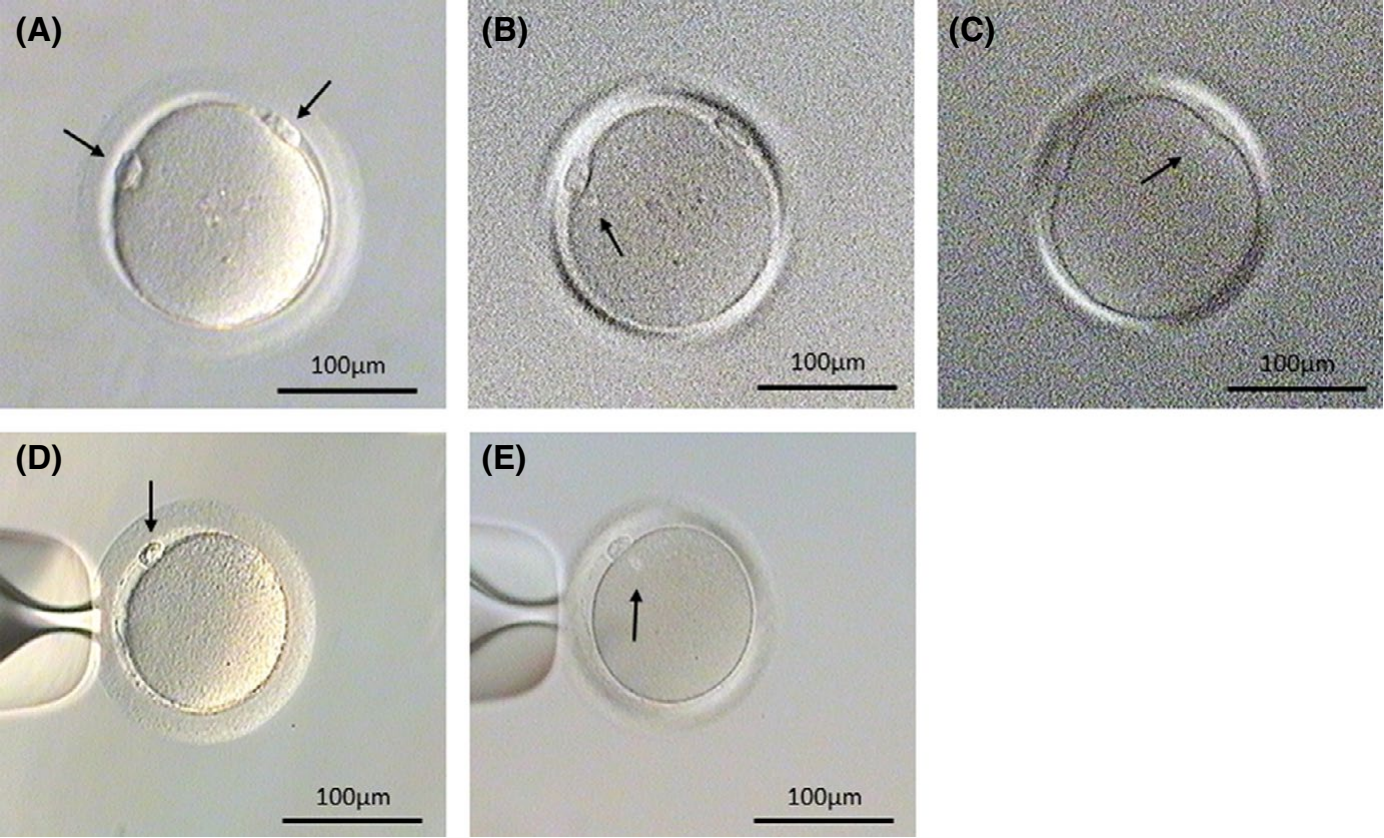
A giant oocyte with two polar bodies and two chromosome‐spindle complexes (CSCs). A, A giant oocyte with two polar bodies (arrows). B & C, A giant oocyte with two CSCs in the cytoplasm. The CSCs (arrows) were observed below the polar bodies by polarized microscopy. D, A normal metaphase Ⅱ oocyte with one polar body (arrow). E, A normal metaphase Ⅱ oocyte with one CSC (arrow)

Ten thousand four hundred and twelve oocytes were obtained between October 2015 and December 2017, in 6129 cycles (clomiphene cycles: 3171; letrozole cycles: 1432; natural cycles: 1526), and the recovered oocytes were included 30 GOs. The GOs recovery rate was 0.29% (30 GOs/10412 oocytes), and the mean of GO cytoplast in diameter was 147.6 ± 0.8 µm. The information on the GOs and donors is summarized in Table 1. In the obtained 30 GOs, the 16 GOs were matured (metaphase II stage) and the 14 GOs were immatured including 6 GOs at metaphase‐I and 8 GOs at germinal vesicle. Of the 16 matured GOs, 13 GOs had 2 CSCs in the cytoplasm (Figure 2) and the other three GOs had one CSC in the cytoplasm. Ovarian cycles in which GOs was obtained were 20 cycles (20 GOs/6606 oocytes, 0.30%) in the clomiphene cycles, 7 cycles (7 GOs/2401 oocytes, 0.29%) in the letrozole cycles, and 3 cycles (3 GOs/1405 oocytes, 0.21%) in the natural cycles, respectively. There was no significant difference (*P* > .05) in GOs recovery rates between patient's ovarian treatment.

**TABLE 1:** Recovered mature giant oocytes (GOs) in the human

No. of GOs	Donor age^a^	Ovarian stimulation method	No. of polar body	No. of CSC(s)^b^	Cytoplasm diameter^c^ of the GOs (µm)
#1	33	Clomid^d^	2	2	147.79
#2	44	Clomid	2	2	145.51
#3	46	Clomid	2	2	144.38
#4	46	Clomid	2	1	148.55
#5	37	Clomid	2	2	153.58
#6	43	Clomid	2	2	143.68
#7	40	Clomid	1	1	153.34
#8	42	Letrozole^e^	2	2	154.21
#9	42	Clomid	1	1	145.20
#10	32	Letrozole	2	2	147.14
#11	43	Natural^f^	2	2	144.78
#12	37	Clomid	2	2	147.76
#13	39	Letrozole	1	1	147.99
#14	43	Clomid	2	2	145.40
#15	43	Natural	2	2	145.71
#16	38	Clomid	2	2	147.27
Mean ± SE	40.5 ± 1.0	‐	1.8 ± 0.1	1.8 ± 0.1	147.6 ± 0.8

^a^ Donor age at oocyte recovery.

^b^ CSC(s): chromosome‐spindle complex(es).

^c^ The cytoplasmic diameter of GOs was measured by RI Viewer^™^ (CooperSurgical, Inc USA).

^d^ Clomid: Oocyte recovery in minimal ovarian stimulation using clomiphene citrate.

^e^ Letrozole: Oocyte recovery in minimal ovarian stimulation using aromatase inhibitor.

^f^ Natural: Oocyte recovery in natural cycle.

### 3.2 GO collection and vitrification

The 16 mature GOs were vitrified and stored in liquid nitrogen. Four of the vitrified GOs were warmed, and all the 4 GOs were survived and used in the present study.

### 3.3 GO micromanipulation for sampling polar bodies and CSCs

**FIGURE 3:**
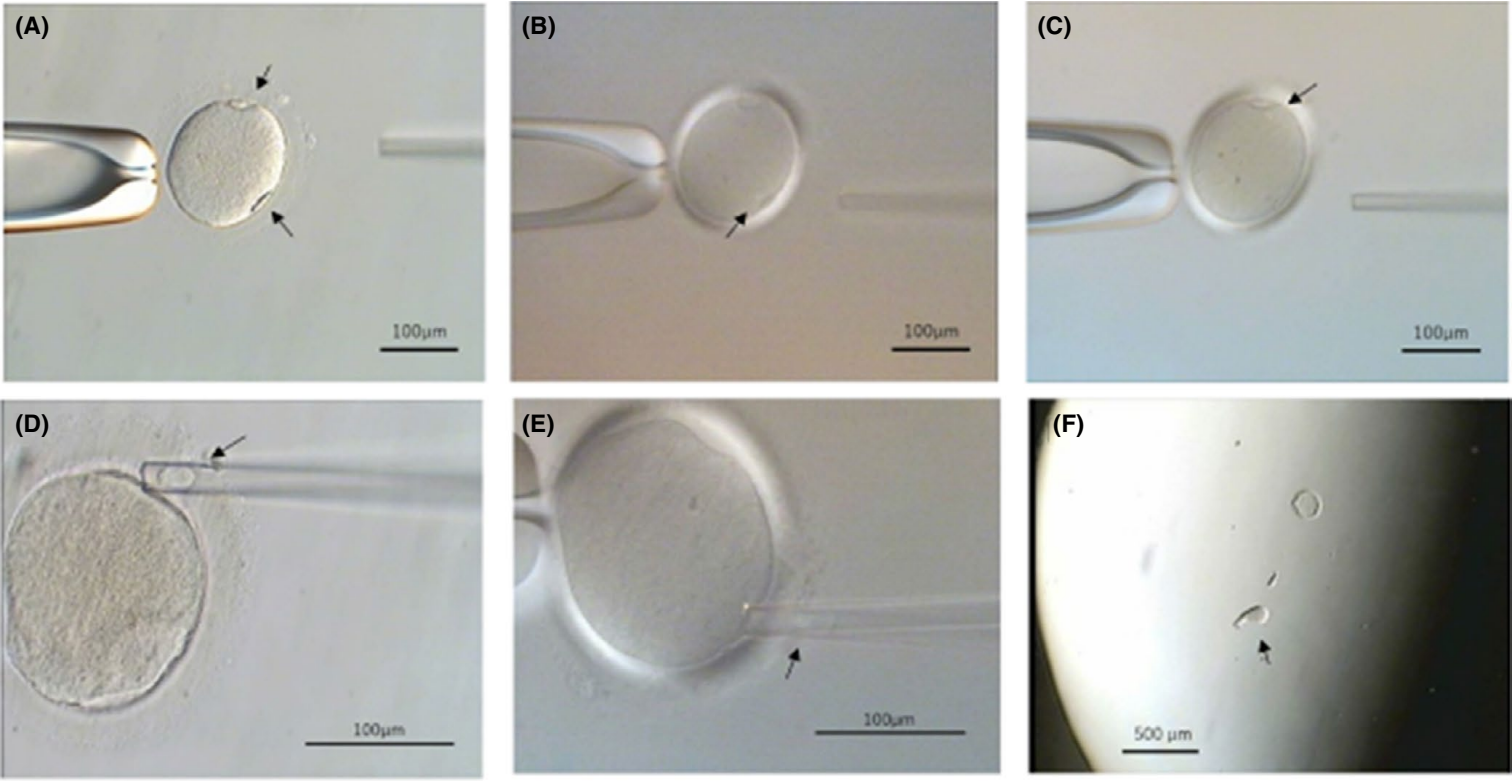
Micromanipulation for sampling of polar bodies, chromosome‐spindle complexes (CSCs), and remained cytoplast from a mature giant oocyte in the human. A, A giant oocyte retained with a holding pipette. The two polar bodies (arrows) are clearly visible by polarized light microscopy. B & C, Confirmation of the two CSCs in the cytoplasm by polarized light microscopy. The CSCs (arrows) were directly observed below the polar bodies. D, Tubing the polar body (arrow). E, Tubing the CSC (arrow) which was observed in the glass pipette. F, A cytoplast (arrow) taken out of the zona pellucida

The successful rate of micromanipulation for sampling (Figure 3) from the GOs was 75% (3/4), and polar bodies and CSCs of the 3 GOs were analyzed by NGS method to examine the chromosome numbers.

### 3.4 Chromosome analyses of GOs by NGS

As shown in Table 2, chromosomal aneuploidy was confirmed by NGS analyses in all the three GOs subjected to polar bodies and CSCs in the GOs (Figure S1‐S3). Comparing the collected spindle genomes with the polar body genomes that was in the vicinity, the deletion and overlapping chromosome numbers were complementary and completely matched on #16 GO (Figure S2) and #11 GO (Figure S3). On the other hand, in #3 GO, duplication of the long arm of chromosome 22 was observed in polar body 1, and monosomy of chromosome 22 was observed in polar body 2, and that CSC 1 showed monosomy of chromosome 3, 5, and deletion of the short arm of chromosome 19, and CSC 2 showed trisomy on chromosome 3 (Figure S1).

**TABLE 2:** Analyses of chromosome numbers in polar bodies and chromosome‐spindle complexes (CSCs) of human mature giant oocytes (GOs) by the next‐generation sequencing (NGS) method

No. of analyzed GOs	Genome derivation of the GOs^a^	Chromosome numbers in CSCs of the GOs^b^
#3	Polar body 1	Aneuploidy: +22q^c^
	CSC 1	Aneuploidy: −3, −5, −19p^d^
	Polar body 2	Aneuploidy: −3, −22
	CSC 2	Aneuploidy: +3
	Cytoplast	Non‐DNA detected^e^
#16	Polar body 1	Aneuploidy: +5
	CSC 1	Aneuploidy: −5
	Polar body 2	Aneuploidy: +7, +14, −20
	CSC 2	Aneuploidy: −7, −14, +20
	Cytoplast	Non‐DNA detected^e^
#11	Polar body 1	Aneuploidy: −15, +17
	CSC 1	Aneuploidy: +15, −17
	Polar body2	Aneuploidy: +16, −17, +19
	CSC 2	Aneuploidy: −16, +17, −19
	Cytoplast	Non‐DNA detected^e^

^a^ The numbers indicate the corresponding combinations.

^b^ +: trisomy, −: monosomy, numbers indicate chromosome number.

^c^ q: the long arm of chromosome, +22q indicates duplication of the long arm of chromosome 22.

^d^ p: the short arm of chromosome, −19p indicates deletion of the short arm of chromosome 19.

^e^ NGS analysis in the present study did not show human chromosome‐specific DNA fragment amplification.

## 4 DISCUSSION

The human GOs recovery rate (0.29%) in the present study was the same as previous reports (0.12%‐0.3%),^1^, ^2^, ^3^, ^4^ and there was no significant difference (*P* > .05) between natural cycles and minimal ovarian stimulation (clomiphene citrate or aromatase inhibitor) cycles. In the present study, comparing the chromosome numbers from CSCs with the chromosome numbers from polar bodies, these chromosomal aneuploidy and structural abnormality were almost complementary in each GOs (Table 2). In #16 GO and #11 GO, chromosomal aneuploidy of the corresponding combinations of polar body and CSC was complementary. Thus, chromosomal aneuploidy presumably occurred during the first meiosis in #16 GO and #11 GO. In #3 GO, chromosomal aneuploidy was partially complementary between polar bodies and CSCs (chromosome 3, 22). Trisomy of chromosome 3 confirmed in CSC 2, and monosomy of chromosome 3 confirmed both polar body 2 and CSC 1. In #3 GO, there seem to be some complementary chromosomes between the polar bodies and between the CSCs, but the reason was unknown. Regarding #3 GO, it seems to have been observed that polar body 2 corresponding to CSC 2 was divided and there were two. In this case, it is probable that the polar body 1 corresponding CSC 1 had disappeared at the time of oocyte recovery. These results suggested that in #16 GO and #11 GO the chromosomal abnormality occurred during the first meiosis.

In the present study, we identified chromosomal abnormalities in the polar bodies of GOs and CSCs of GOs (Table 2). The chromosomal abnormalities observed were monosomy and trisomy, and tetrasomy was not observed. The increase in miscarriage rates in older pregnancies is largely due to chromosomal aneuploidy that occurs during the first meiosis.^13^ It has been reported that this is one of the causes of the decrease in cohesin in the oocyte chromosome with aging,^14^ and it is thought that this is because the decrease in cohesin causes the early separation of bivalent chromosomes.^15^

It is considered that the chromosomal abnormalities in GOs observed in the present study might be due to the early separation of bivalent chromosomes. The age of patients whom GOs was used in the present study was 46 for # 3, 43 for # 11, and 38 for # 16, respectively, and it is undeniable that the age affected chromosomal abnormalities of the GOs. In addition, it is considered that GOs are highly likely to be cells that are prone to chromosomal abnormalities due to their structural characteristics.

About the chromosome analysis by NGS method applied in the present study, there is concern that the genomic amount of the analysis sample is smaller than general samples. In PGT‐A in human blastocysts, considering the possibility that abnormal cells are contained, a method to collect and analyze 5‐10 cells at once is recommended.^16^ Since the NGS analysis used in the present study was a set of chromosomes included in each first polar body and M II phase CSC, one of the problems was whether the whole genome amplification process required for NGS analysis succeeded it was. But analysis accuracy was sufficient from the viewpoint of lead number, and median of the absolute values of all pairwise differences (MAPD) score was <0.45.^17^

The results of the present study showed that CSCs and corresponding polar bodies of GOs could be collected as genomic samples by micromanipulation and that CSCs and the polar bodies could be analyzed by NGS method. Furthermore, it was revealed that human GOs should not be oocytes targeted for ART, as the number of chromosomes was abnormal. However, as the present study was limited to the analysis of 3 GOs, it is necessary to further increase the number of chromosome analysis of GOs.

In conclusion, we could collect the CSCs and the polar bodies from human GOs by micromanipulation, and then could analyze the chromosome numbers of the GOs by NGS method. As our data suggest that human GOs have chromosomal abnormalities including aneuploidy, GOs should be excluded from clinical purpose as gamete sources for embryo transfer in the human.

## DISCLOSURES

*Conflict of interest*: The authors declare no conflict of interest in association with this manuscript. *Human rights statement and informed consent*: The present study was approved by the institutional ethics review board of Yamashita Shonan Yume Clinic held in October 2015 (Reference number: YSYC‐07) and National Center for Global Health and Medicine Ethics Review Board held in February 2020 (Reference number: 3370). All patients signed an informed written consent form before entering the study, and they were informed that they could terminate their cooperation with us whenever they wanted without any consequences.

## Supporting information

Fig S1‐S3
