# Supplementary material for: Chromosomal analyses of human giant diploid oocytes by next‐generation sequencing
Source: Reprod Med Biol. 2021 Mar 18;20(3):260–6. doi: 10.1002/rmb2.12378 (PMC8254173; doi:10.1002/rmb2.12378)
Supplement: Supplementary file 1 — Fig S1‐S3 [file RMB2-20-260-s001.pdf]

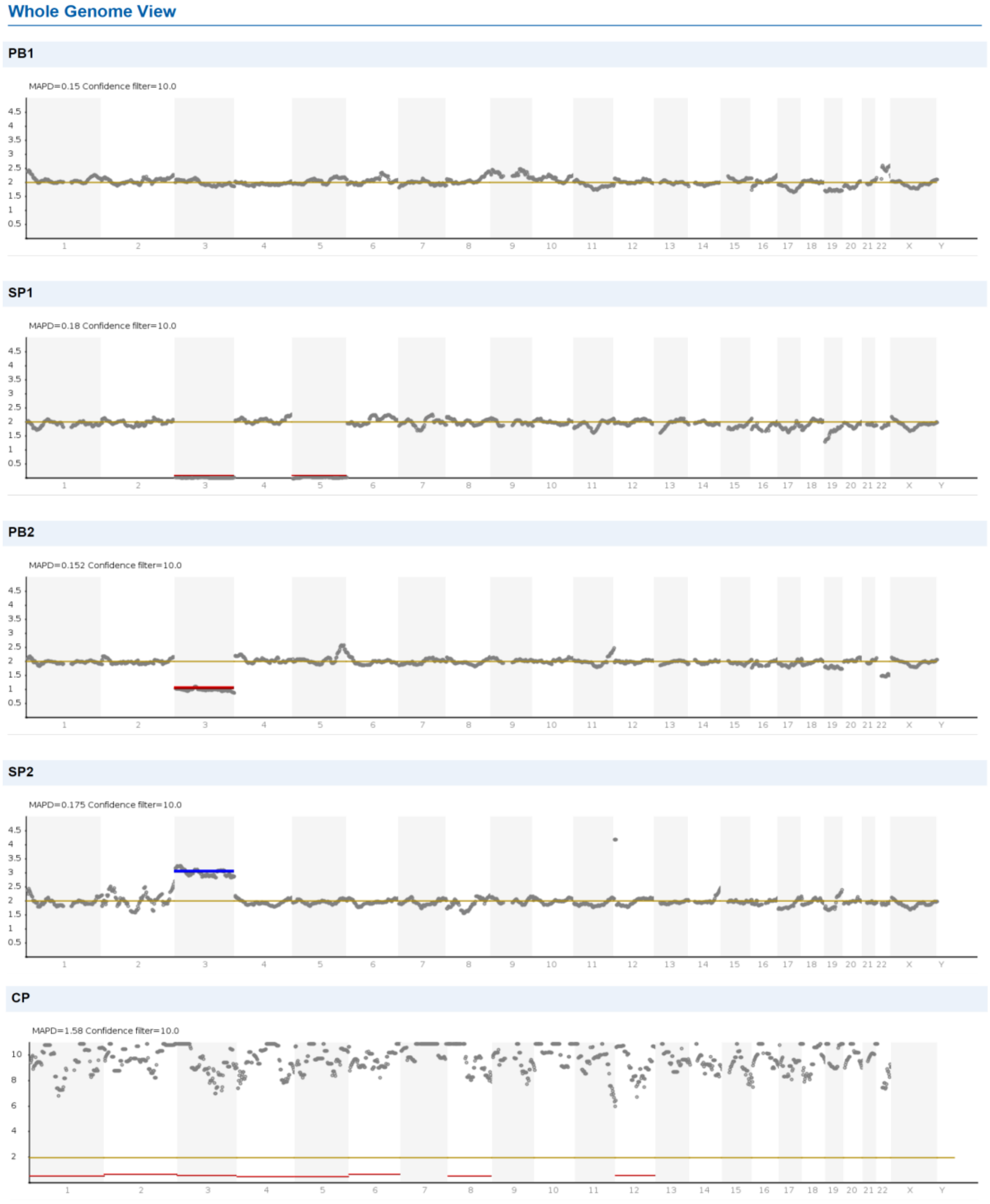

Figure S1

Whole genome view as relative value of each chromosomes derived from the #3 giant oocyte (GO) by next-generation sequencing analyses

PB indicates a polar body-derives genome, SP indicates a CSC-derived genome, and CP indicates a cytoplast-derived genome. The numbers after PB and SP indicate that they correspond to each other. The numbers and letters (1 to 22, X, Y) at the bottom of the graph indicate the corresponding chromosome numbers and sex chromosomes. The vertical axis of the graph shows the amount of amplification of the genome, and the value of 2 is euploid. One (1) indicates monosomy and 3 indicates trisomy. The results are shown in Table 2.

CSC: Chromosome spindle complex

Citation: Visualization Report, Igenomix Japan K.K.

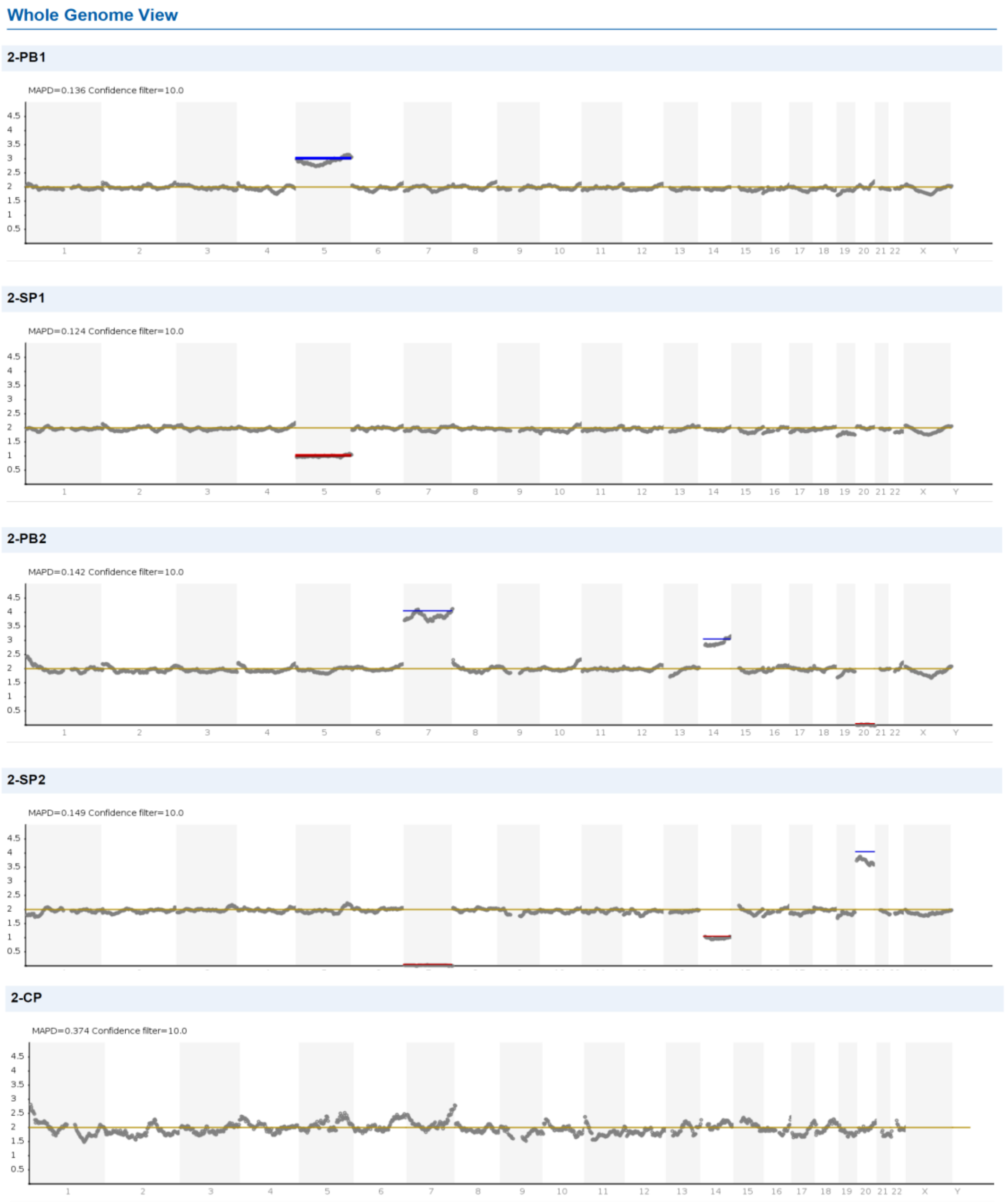

Figure S2

Whole genome view as relative value of each chromosomes derived from the #16 giant oocyte (GO) by next-generation sequencing analyses

PB indicates a polar body-derives genome, SP indicates a CSC-derived genome, and CP indicates a cytoplast-derived genome. The numbers after PB and SP indicate that they correspond to each other. The numbers and letters (1 to 22, X, Y) at the bottom of the graph indicate the corresponding chromosome numbers and sex chromosomes. The vertical axis of the graph shows the amount of amplification of the genome, and the value of 2 is euploid. One (1) indicates monosomy and 3 indicates trisomy. The results are shown in Table 2.

CSC: Chromosome spindle complex

Citation: Visualization Report, Igenomix Japan K.K.

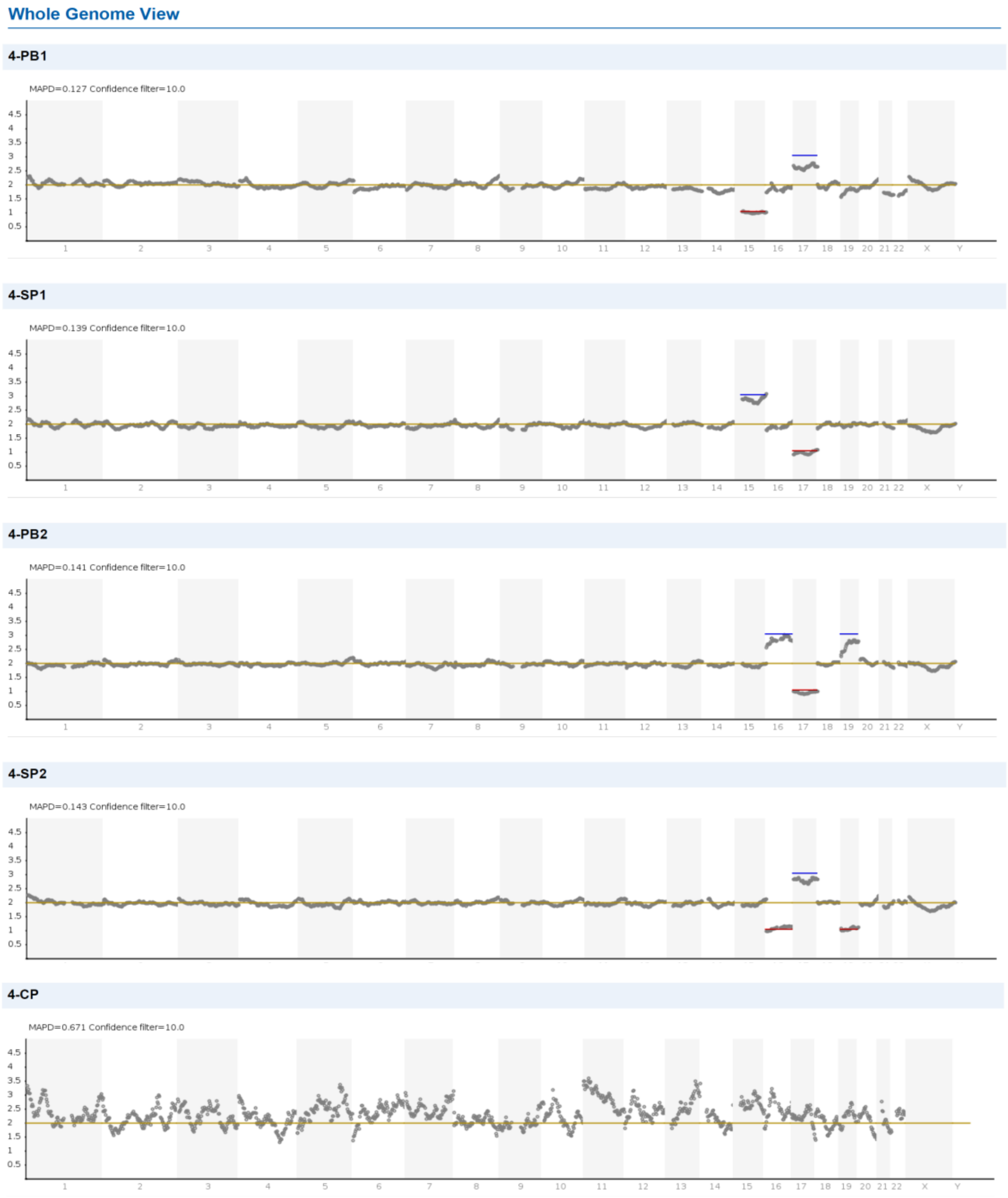

Figure S3

Whole genome view as relative value of each chromosomes derived from the #11 giant oocyte (GO) by next-generation sequencing analyses

PB indicates a polar body-derives genome, SP indicates a CSC-derived genome, and CP indicates a cytoplast-derived genome. The numbers after PB and SP indicate that they correspond to each other. The numbers and letters (1 to 22, X, Y) at the bottom of the graph indicate the corresponding chromosome numbers and sex chromosomes. The vertical axis of the graph shows the amount of amplification of the genome, and the value of 2 is euploid. One (1) indicates monosomy and 3 indicates trisomy. The results are shown in Table 2.

CSC: Chromosome spindle complex

Citation: Visualization Report, Igenomix Japan K.K.
